# Aortic Valve Replacement Via Right Anterior Mini-Thoracotomy: the Conventional Procedure Performed Through a Smaller Incision

**DOI:** 10.21470/1678-9741-2020-0165

**Published:** 2021

**Authors:** Gabriele Tamagnini, Raoul Biondi, Mauro Del Giglio

**Affiliations:** 1 Department of Cardiac Surgery, Villa Torri Hospital, Bologna, Italy.

**Keywords:** Aortic Valve, Thoracotomy, Mammary Arteries, Heart Valve Prosthesis, Prothesis Implantation, Drainage, Catheterization

## Abstract

Minimally invasive aortic valve replacement has gained consent due to its good results in terms of minimized surgical trauma, faster rehabilitation, pain control and patient compliance. In our experience, we have tried to replicate the conventional and gold standard approach through a smaller incision. Sparing the right internal thoracic artery, avoiding rib fractures and performing total central cannulation is important to make this procedure minimally invasive from a biological point of view too. In addition, the total central cannulation is pivotal to simplify perfusion and drainage. Moreover, a complete step-by-step procedure optimization and-when possible-the use of sutureless prosthesis help to reduce the cross-clamping and perfusion times. After more than 1000 right anterior thoracotomy (RAT) aortic valve replacements, we have found tips and tricks to make our technique more effective.

## INTRODUCTION

Minimally invasive aortic valve replacement (AVR) via right anterior thoracotomy (RAT) (MiAVR-RAT) is a technique developed in the past few years. There were some concerns due to the limited exposure of anatomical structures, which makes this procedure more challenging. Several published studies comparing AVR through RAT and/or upper mini-sternotomy *versus* full sternotomy report longer cardiopulmonary bypass (CPB) and cross-clamp times in the non-conventional approaches^[1]^. Prospective randomized studies have shown advantages of the minimally invasive approach in terms of decreased bleeding, post-surgical pain and trauma, and shorter hospital and intensive care unit (ICU) stay times, with consequent costs reduction^[2-4]^. Moreover, with the advent of transcatheter aortic valve implantation (TAVI), there is an interest in minimizing the trauma of surgical AVR^[5]^. In our experience, the advantage of a minimally invasive approach expands well beyond the benefit of a better cosmetic result. 

## TECHNIQUE

Pre-operatively, we did not perform any computed tomography (CT) scan for patient selection; we believe it might be a useful tool only in the first phase of the learning curve, to avoid cases of very deep or left-sided aorta, very calcified aorta and to address the right place for the surgical access. 

**Fig. 1:**
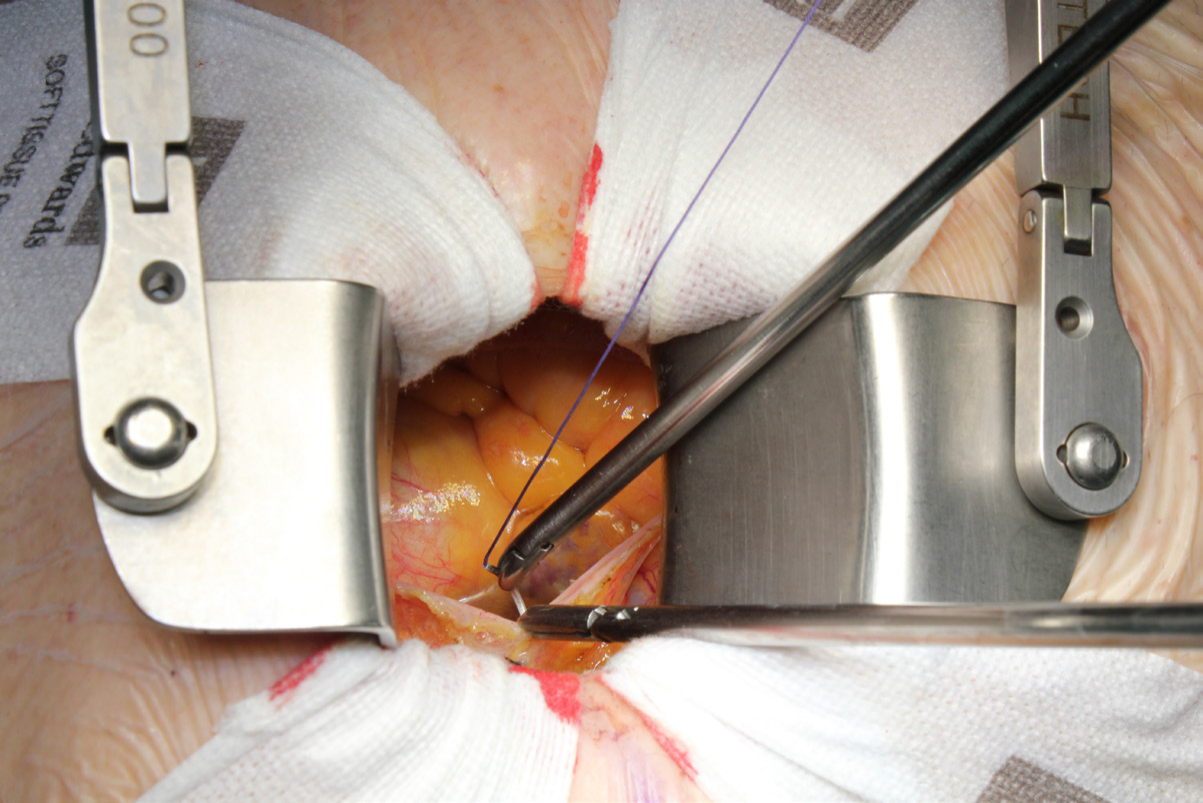
Surgeon's view: opening the pericardium.

**Fig. 2:**
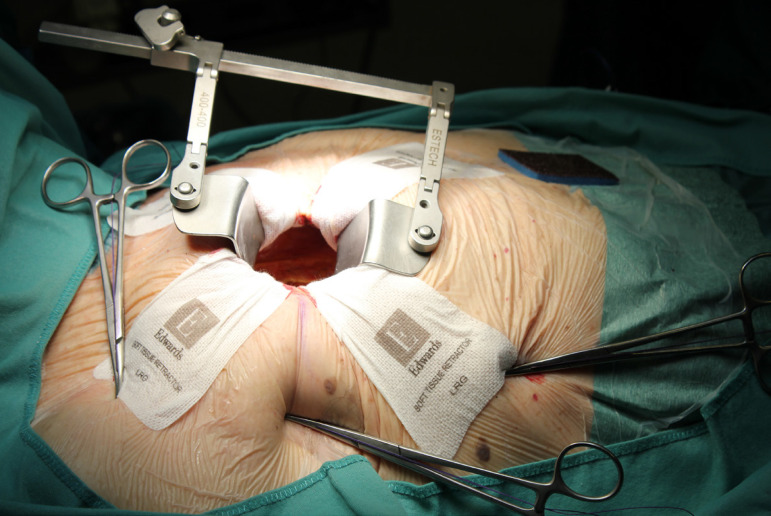
The three pericardial retraction stitches through the thoracic wall.

**Fig. 3:**
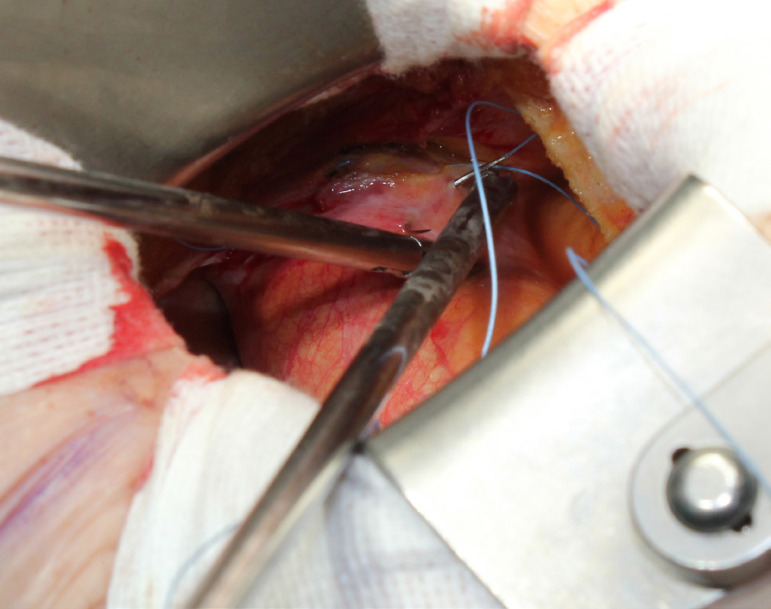
Aortic purse-string sutures at the level of the pericardial reflection.

**Fig. 4:**
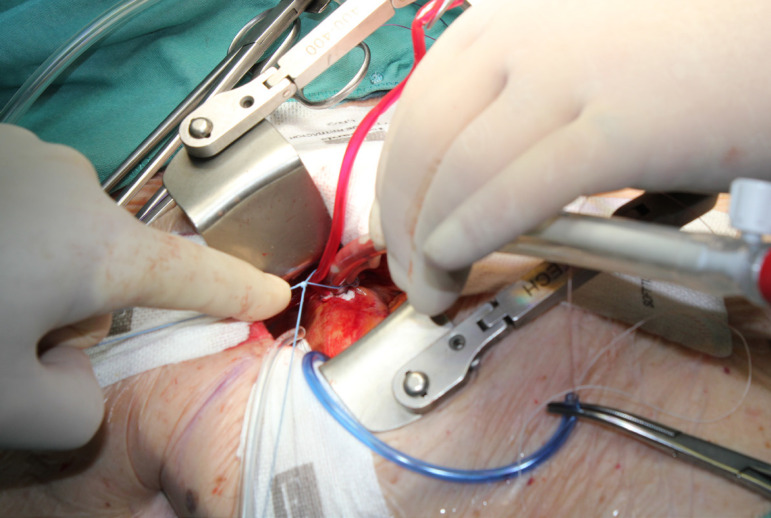
Aortic cannulation: handier than it may seem.

**Fig. 5:**
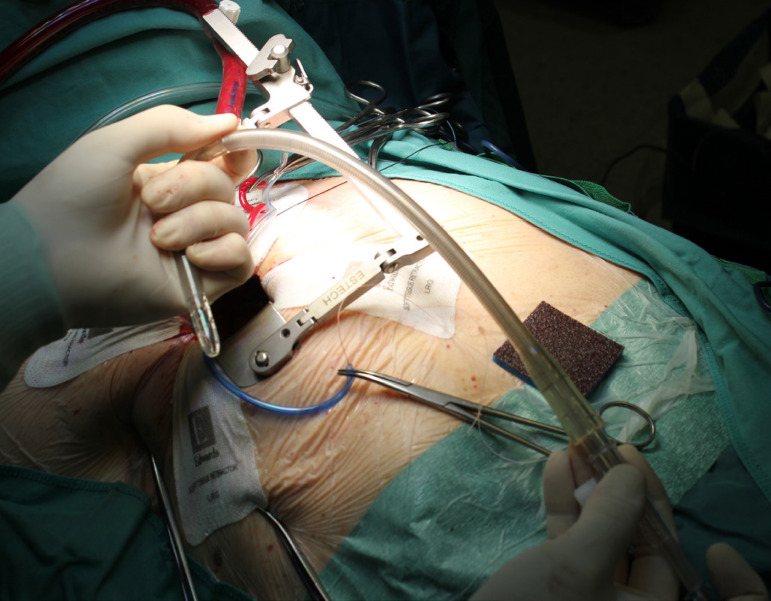
Bending the venous cannula with a metal spindle makes it easier to engage the inferior vena cava.

**Fig. 6:**
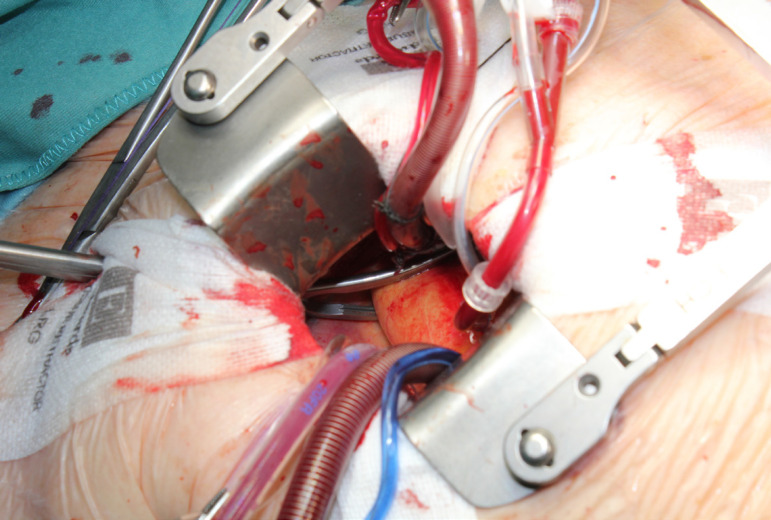
Aortic cross-clamping with a Chitwood- DeBakey clamp.

**Fig. 7:**
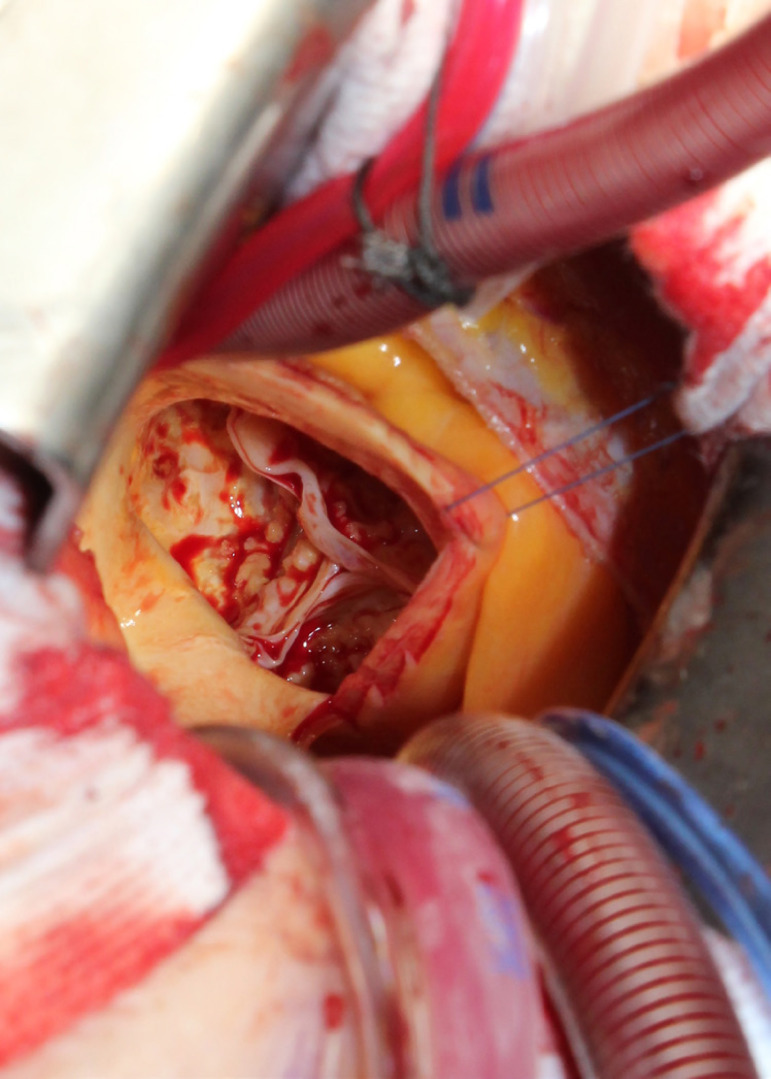
Diseased aortic valve from the surgeon's spot: we performed this operation in direct view

**Fig. 8:**
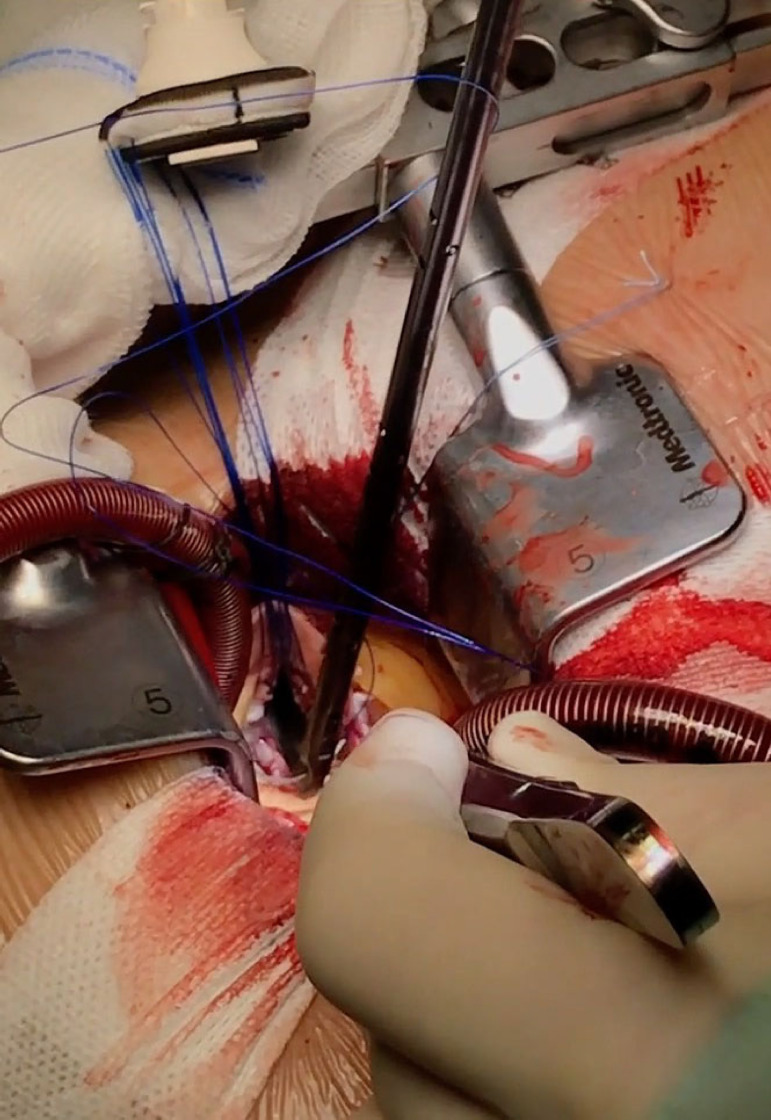
In sutured prostheses, we prefer to implant the valve with 3 running sutures.

The patient lies in supine position, with an inflatable bag behind the right scapula to tilt the thorax towards the left side. Double-lumen intubation is provided to allow exclusion of the right lung. A 5-cm skin incision is made following the line of the third rib and the second or third right intercostal space is opened, respecting the right internal thoracic artery and avoiding any rib damage. To identify the correct intercostal space, we have come up with a kind of "rule of thumb": we divided the linear distance between the right clavicle and the ipsilateral costal arch into equal quarters and then performed the incision in the middle of the second quarter. In our experience, that point accounts for the best view: ideally, the right superior pulmonary vein should be exactly in the center of the surgical field. Remember that it is easy to change space from the same skin incision if the anatomical variance offers a poor exposition. A soft tissue retractor and a spreader are used to help to spread the intercostal space. The pericardium is opened as far away from the phrenic nerve as possible, almost as in the median sternotomy. Ideally, the incision will run-up to the pericardial reflection and down towards the diaphragm, to expose the right atrium as much as possible. It is important to pay attention to do a proper hemostasis of the pre-pericardial fat. We use three pericardial retractions, making a deep figure-of-eight suture with a strong, thick wire (Figure 1): the first at the level of the right superior pulmonary vein, the second as cranial as possible (next to the pericardial reflection in the aorta) and the last as low as possible; these stitches are passed through the thoracic wall with an Endo Close (Covidien) device (Figure 2). Thanks to these retractions, the exposure can be optimized: first, the heart will be taken to the surgeon and, second, the field could be moved cranially or caudally. Our technique strictly relies on total central direct arterial and venous cannulation: for arterial cannulation, an EOPA (Elongated One-Piece Arterial Cannula, Medtronic) cannula is inserted through a double purse-string suture placed in the proximal part of the aortic arch or in the pericardial reflection on the aorta (Figures 3 and 4); a three-stage MC2X cannula (Medtronic) is placed in the right atrium. For the arterial cannulation, it is crucial to obtain an optimal view and control of the target spot: a useful trick is pushing the aorta down and towards you with a sponge and forceps. For the venous cannulation, as it might be difficult to engage the inferior vena cava with a straight cannula, we use a metal spindle (*e.g*. the one included in the intracavitary venting small cannula) to give a rounded shape to the venous one (Figure 5). Moreover, we use a snare strongly tied around the venous cannula and the tourniquet next to the right auricula, then we pass that through the thoracic wall to pull the right atrium away and obtain a better aortic root exposure. The left ventricle is vented as in routine procedures, thanks to a small cannula inserted through the right upper pulmonary vein. Continuous insufflation of carbon dioxide is used during the operation at a flow rate of 3 L/min. The aorta is cross-clamped at the origin of the innominate artery with a Chitwood DeBakey clamp, without freeing up the aorta from the pulmonary artery. To achieve a less crowded operative field, the clamp is inserted through a separate skin incision of less than 1 cm, just below the lateral part of the clavicle, and is placed in the transverse sinus: we use the suction cannula to make enough space and to avoid any injury to blood vessels (Figure 6). Cold blood cardioplegia is administered in an antegrade fashion. Normothermic CPB is performed in all patients. A transverse incision of the ascending aorta is performed (Figure 7), and native valve leaflets are excised *en bloc*. We implant either sutureless or sutured prostheses. In sutured prostheses, the technique of choice is to use three 2-0 Prolene running sutures (120 cm) with 3 or 4 big steps for each sinus (Figure 8): this way, in our experience, we have almost no paravalvular leakage, better hemodynamic performance and faster implantation. To properly stretch the sutures, we use a silk thread in the middle of the suture for countertraction. The de-airing process is done by gently filling the heart. The ascending aorta incision is closed with a single or double 4-0 Prolene suture, depending on the quality wall. The aortic clamp is removed and the patient is weaned from CPB. The ventricular pacing wires are placed on the right ventricle. The cannulae are removed and protamine is administered. Hemostasis is crucial when working in a narrow field; hence, we use polytetrafluoroethylene (PTFE) pledgets on every purse-string. 

## DISCUSSION

After performing more than 1000 aortic valve replacements via right anterior mini-thoracotomy, we have come up with advice.

First, the use of central cannulation reduces surgical trauma and avoids potential complications due to femoral cannulation, allowing a more physiological perfusion flow and better venous drainage. Indeed, peripheral cannulation and, therefore, retrograde arterial flow-often chosen by other minimally invasive specialized teams-have been reported to correlate with dissection of the femoral vessel and aorta, ipsilateral limb ischemia, cerebrovascular and renal events^[6]^. From a technical point of view, the possibility of using a properly sized arterial cannula (instead of a smaller femoral option) is pivotal to reduce the shear force during the CPB time. 

Second, we would enlighten the tight link between operative time and "biological" invasiveness: the longer the cardioplegic arrest and the CPB time, the greater the biological cost the patient would pay^[7]^. One of the most heard criticisms of the minimally invasive approach is, indeed, the broader need for time to operate and we do support this idea: the minimally invasive approach could not be justified in exchange for operative time, by any means. On the contrary, the minimally invasive technique must imply optimized steps to smoothen and fasten the whole procedure. For example, we place some retraction stitches only after removing the native valve and dimensioning the prosthesis; usually, we start closing the aortotomy while the nurse prepares the prosthesis; we perform a full de-airing process, asking the perfusionist to slowly fill the heart while finishing the closure of the aorta. Surely, the use of a sutureless solution helps to speed up the procedure: at the beginning of the experience, it might help to balance out the time the surgeon would spend to build-up his or her expertise.

Third, when using a sutureless prosthesis, the permanent pacemaker implantation rate is a hot topic. If the Perceval valve bioprosthesis is too deep in the outflow tract, we believe there would be a stronger radial force straight to the membranous septum, with interference in the bundle of His. For this reason, we place that prosthesis as high as possible, but respecting the implant recommendations. In an in press paper, we reported a patient-prosthesis mismatch (PPM) rate of 2,3%; if we look at the available data on the need for permanent pacemaker in Perceval S series, we find rates ranging from 8 to 23%; after having adopted surgical tricks, the authors found a PPM rate of 4.3%^[8]^.

Forth, in our experience, the advantages of this surgical technique include early mobilization and a shorter rehabilitation period, less postoperative pain, good cosmetic results with a lower incidence of wound complications, especially in high-risk patients (obese, diabetic, or osteoporotic ones). After a 10-year experience with RAT-AVR, we use this technique systematically, regardless of the body mass index and the shape of the thorax. In terms of nurse compliance, our technique is comparable to the standard approach: we tried to keep the usual workflow, without jeopardizing the nurse's expertise in conventional surgery. All the materials, CPB machine, and cannulas we use are the same as in full sternotomy operations. Some minimally invasive instruments are the only additional tools we require.
